# The Influence of External Sulfate Attack on the Durability of Reinforced Mortars in the Presence of Calcined River Sediments

**DOI:** 10.3390/ma16206684

**Published:** 2023-10-13

**Authors:** Ali Benkabouche, Mouhamadou Amar, Mahfoud Benzerzour, Nor-Edine Abriak, Michèle T’kint, Mohamed Mouli

**Affiliations:** 1 IMT Nord Europe, Institut Mines-Télécom, Centre des Matériaux et Procédés, 59000 Lille, France; ali-zine.benkabouche@imt-nord-europe.fr (A.B.); mahfoud.benzerzour@imt-nord-europe.fr (M.B.); nor-edine.abriak@imt-nord-europe.fr (N.-E.A.); 2 Research Unit Eco-Processes, Optimization, and Decision Support, Picardie Jules Verne University, 7 Street Moulin Neuf, 80000 Amiens, France; michele.tkint@u-picardie.fr; 3 Department of Civil Engineering, Laboratory of Materials, ENPO, Oran 31000, Algeria; moulimohamed@yahoo.fr

**Keywords:** calcined sediments, external sulfate attack, corrosion potential

## Abstract

In France, the annual volume of dredged sediments is significantly increasing, which has become a real environmental problem. Nevertheless, these sediments can be used beneficially as supplementary cementing material. On the other hand, external sulfate attack is one of the most aggressive causes of deterioration that affects the durability of concrete structures. This study focused on the valorization of river-dredged sediments from Noyelles-Sous-Lens (Hauts-de-France) as a mineral addition in substitution of Portland cement, and it studied their impacts on the mechanical behavior and durability of reinforced mortars. X-ray diffraction (XRD) analysis indicated the presence of clay minerals in the raw sediment. In order to activate this clay fraction, flash calcination was applied at a temperature of 750 °C. In addition, four mixed mortars were formulated by mixing a Portland cement (CEM I 52.5 N) and the calcined sediments as a partial substitute for cement with proportions of 0%, 15%, 20%, and 30%, then stored in water tanks at room temperature (20 ± 2 °C) for 90 days in order to immerse them in a tank containing a 5% MgSO_4_ solution and to track the evolution of their corrosion potential as well as their mass variations every 20 days for a period of 360 days. The following additional tests were carried out on these mortars: tests of resistance to compression and flexion and to porosity by mercury intrusion. The results obtained from the majority of these tests showed that the mortar containing 15% calcined sediments is as effective and durable as the reference mortar itself. The main conclusion we can draw from these results is that the presence of these calcined sediments improves the overall behavior of the mortar.

## 1. Introduction

The demand for natural resources has greatly increased over the last century, especially in the field of civil engineering, since all construction or rehabilitation projects require the use of granular materials. Consumption amounted to 453.1 million tons in France in 2019, with 72.3% of this total consisting of natural aggregates and 27.7% recycled aggregates (ERMCO, 2019) [1].

Cement is one of the most commonly used construction materials in the world. According to ERMCO, the production of concrete in France reached 44.4 million m^3^ [1]. Massive increases in construction and industrialization have created huge demands for cement and concrete production [2]. Cement production is a process that requires natural resources and energy. About 50% of the CO_2_ is then attributed to the cement production process [3], and this production leads to the emission of more than 2.3 gigatons of CO_2_ worldwide [4].

One strategy to reduce the industry’s carbon footprint is the use of blended cement, which partially replaces ordinary Portland cement with supplementary cementitious materials (SCMs) that are also called mineral supplements. Instances of these materials are fly ash, which is a by-product of coal-fired power plants, and ground granulated blast-furnace slag obtained as waste from the steel industry [5]. Currently, the average clinker/cement ratio is about 73.7% [6], but the potential for further reduction is limited as the supply of the most desirable high-value SCMs (fly ash and blast-furnace slag) decreases [7,8,9]. This demonstrates the need for new alternative sources of SCM.

The use of calcined clays as pozzolanic materials in concrete has received considerable interest in recent years as part of a broader focus on the use of waste materials, locally available minerals, and industrial by-products as alternative cement additives [10,11]. Calcined dredged sediments, which initially contain substantial amounts of clay minerals, are promising new SCMs for use in concrete [12,13,14,15,16,17,18,19,20,21]. A large amount of dredged sediment is accumulated. Every year, a total of 56 million m^3^ is dredged from the sea and river bottoms in France. Currently, most of the dredged sediments are dumped at sea or deposited in landfills, which has a negative impact on the environment [22]. The beneficial reuse of these dredged materials as secondary raw materials can therefore provide a sustainable solution to limit landfill waste and limit the extraction of natural resources [17]. The objective of this paper is to study the influence of the valorization of sediments in reinforced mortars and their beneficial uses.

Several researchers are already studying the valorization of dredged sediments for their use as raw material [23], either to replace part of the raw material in the manufacture of Portland cement [24,25] or as a mineral addition for concrete [26,27]. Most studies focused on the characterization of dredged sediments and their behavior in cement paste and mortar [12,14,16,17,18]. The properties, pozzolanic reactivity, and hydration processes of calcined dredged sediments were intensively studied by the authors [28,29]. Other researchers [30] worked on low-carbon and calcined-sediment-based binders as sustainable construction materials. They demonstrated that by substituting calcined sediments at 5%, 15%, and 25% for cement, the climate change potential indicator (which measures the potential greenhouse gas emissions) improves. Specifically, using calcined sediments at these percentages leads to a reduction of 10.70%, 17.84%, and 3.57%, respectively, compared to using 100% cement. The emissions for each substitution rate are 901.73 kg CO_2_ eq/T, 835 kg CO_2_ eq/T, and 768.29 kg CO_2_ eq/T.

However, the durability of materials developed from sediments has been studied only very briefly [26,27,31,32,33]. According to Amar [26], after studying the effect of the partial replacement of cement with sediment calcined at a temperature of 850 °C for 1 h, the results of the tests highlighted the fact that the mortar containing 10% treated sediment is as effective and durable as the reference mortar itself. It is deduced that the presence of these calcined sediments improves the overall behavior of the mortar. In addition, Achour [27] found that the optimal percentage of sediment replacement by aggregate is 12.5% in order to maintain the integrity, strength, and resistance of the concrete to chemical attack and frost action. From the perspective of this paper, the durability of mortars based on sediments is studied in order to develop the latter’s capacity to resist physical–chemical attacks while maintaining its desired mechanical properties. Tests of compressive strength and flexural strength, mercury porosity, external sulfate attack, and corrosion potential (E) were conducted on these mortars.

### 1.1. Materials and Methods

In this section, we will discuss the materials and equipment used during this research work.

### 1.2. Flowchart 

**Figure 1:**
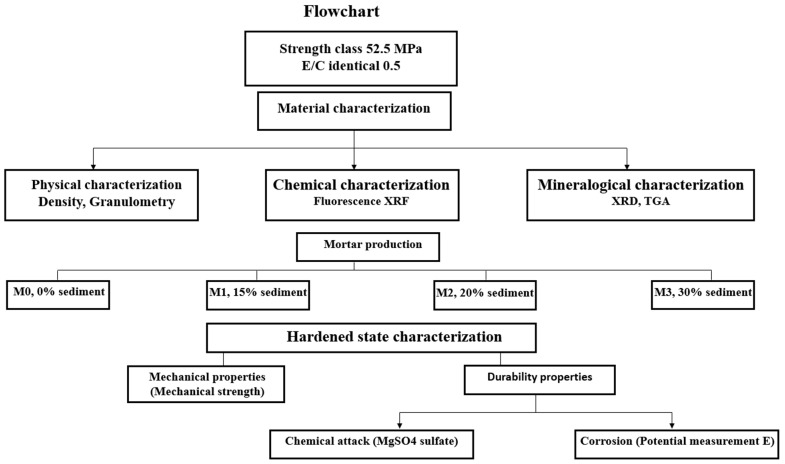
Flowchart summarizing the experimental work carried out in this article.

Figure 1 show flowchart summarizing the experimental work carried out in this article.

### 1.3. Materials

The river sediment used in this study collected from Noyelles-Sous-Lens in northern France. After reception at the laboratory, it was homogenized, quartered, and then dried in an oven at 105 °C to constant mass to remove moisture. It was then ground using a flail mill with a 250 μm grate to achieve a particle size of less than 250 μm before flash calcination. The sediment was calcined at a temperature of 750 °C. The raw and calcined sediments are named below RS and SF 750, respectively.

The cement used was an ordinary commercial Portland CEM 52.5 N (referred to as OPC) according to the NF EN 196-1 standard [34]. 

The steel bars used in the manufacture of cylindrical reinforced mortars were ribbed, with high adhesion and measuring 8 mm in diameter.

The sand used was certified according to NF EN 196-1 [34], with a grain size distribution between 0.08 and 2 mm. Table 1 contains the nomenclature of the materials in this study.

### 1.4. Calcination of Sediments: “The Process”

**Figure 2:**
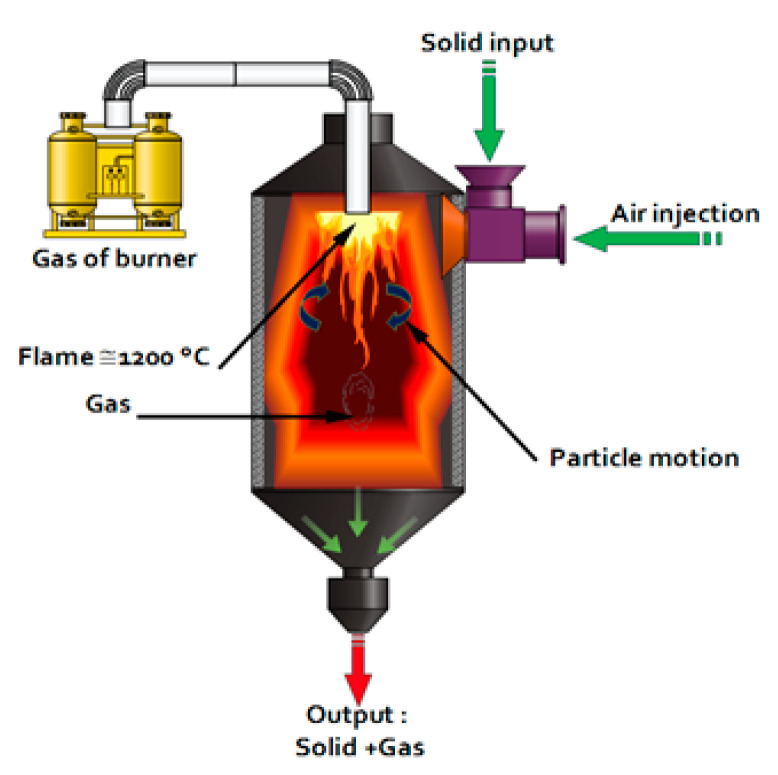
Flash calcination system.

The calcination method used was flash calcination. Flash calcination is a heat treatment technique consisting of the rapid exposure of finely divided materials in the presence of air at high temperatures [35]. The flash calcination technique was initially used to chemically activate certain clays such as kaolinite in order to give them pozzolanic properties. When this technique was applied to specific categories of clays such as kaolins, dehydroxylation following a dehydration process was noted [36]. This corresponds to the removal of a hydroxyl bond (OH^−^) occurring between 450 and 750 °C [37]. Flash calcination leads to a partial destructuration of the material, a state of amorphization, and thus, a potential reactivity of the material [38]. The diagram of the calcination unit and the principle of the pilot are shown in Figure 2.

### 1.5. Characterization Tests

All materials used were characterized by physical and chemical methods. The determination of the granulometry was carried out with a COULTER LS13320 (Beckman Coulter, Brea, CA, USA). This laser device enables the distribution of granular particles below 1 µm to be determined. The BET (Brunauer–Emmett–Teller) method (NF EN 196-6 [39]) is an estimation of the surface area to assess fineness (NF EN ISO 18757 [40]) using an Autopore IV 9505 from Micromeritics (Norcross, GA, USA). The organic fraction was also determined. The specific gravity of the materials was determined via an AccuPyc1330 helium pycnometer from MICROMETRICS according to NF EN 1097-7 [41]. The porosity of mercury was also measured using an Autopore V 9600 porosimeter (Micromeritics Instrument Corporation, Norcross, GA, USA) according to the standard NF P 94-410-3 [42]. TGA analysis is a thermal analysis method that follows the mass loss of a sample as a function of time or temperature in a controlled atmosphere. It was performed on a NETZSCH STA 409 (Waldkraiburg, Germany) apparatus using nitrogen gas, with a ramp of 10 °C/min and a temperature range of 40 to 1000 °C. The chemical composition was measured by X-ray fluorescence analysis (XRF) according to the NF EN 196-2 standard with a PIONEER S4 equipped with a 4 kW generator and a rhodium anode.

Mineralogical characterization by X-ray diffraction (XRD) analysis was performed using the apparatus (XRD Bruker D2 advanced device equipped with Cu k*α*. *λ* = 1.5406 Å) with an acquired angle of 2θ from 5° to 80° and a 0.02 step. This identified the mineralogical nature and crystalline phases present and was performed with a setting of 40 kV and 40 mA. This method was formerly used by several authors in the study of mortar-based sediments [43,44,45]. Bending and compressive strength tests were performed using three 4 × 4 × 16 cm^3^ prismatic samples (NF EN 196-1 [34]).

Cubic mortar specimens 5 × 5 × 5 cm^3^ and cylindrical reinforced mortars 11.2 × 5 cm^2^ were prepared and cured in water for 90 days and then partially immersed in a solution of MgSO_4_ for 360 days according to ASTM C1012-04 (2004) [46] in order to evaluate the effect of sulfate attack on the hardness of these mortars. Firstly, the monitoring of the mass variation, evolution of the compressive strength, and visual analysis of the condition of the specimens were carried out on the cubic mortars. Then, the evolution of the corrosion potential of the cylindrical mortars was measured using a voltmeter and a Cu/CuSO_4_-saturated copper-saturated Cu/CuSO_4_ copper sulfate electrode.

## 2. Results

### 2.1. Physical Properties

The physical properties of the materials used are presented in Table 2. OPC has the highest density with 3.15 g/cm^3^, followed by SF 750 with 2.65 and RS with 2.48, respectively. SFC has the highest specific surface (27.49 m^2^/g).

### 2.2. Granulometry

**Figure 3:**
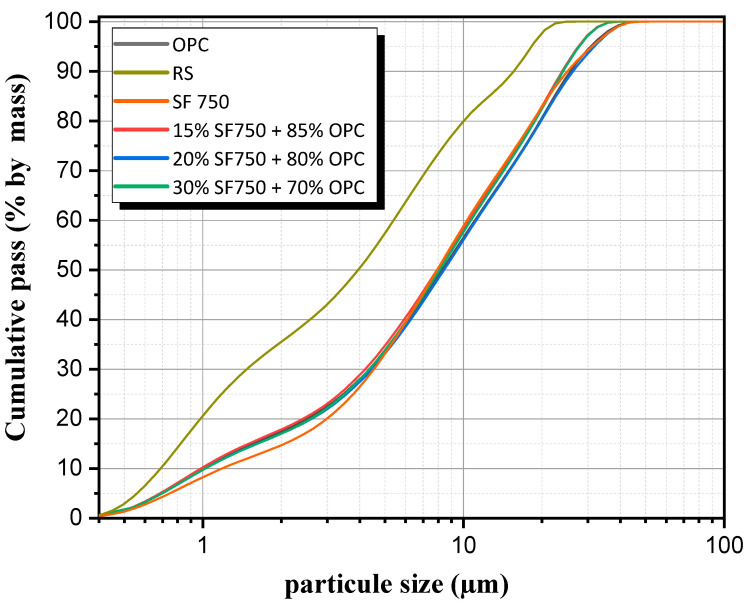
Granulometric analysis.

The particle size results are presented in Figure 3. The results show that the calcined flash sediment (D90 = 25 μm) has a coarser particle size than the raw sediment (D90 = 15 μm). The calcination contributes to the sintering of the particles, which thus forms larger particles. However, some of these particles may have internal nanoporosity, which allows for increased reactivity [28]. For cement-based binders and sediments, the particle size is almost the same as the individual materials.

### 2.3. Thermogravimetric Analysis (TGA)

**Figure 4:**
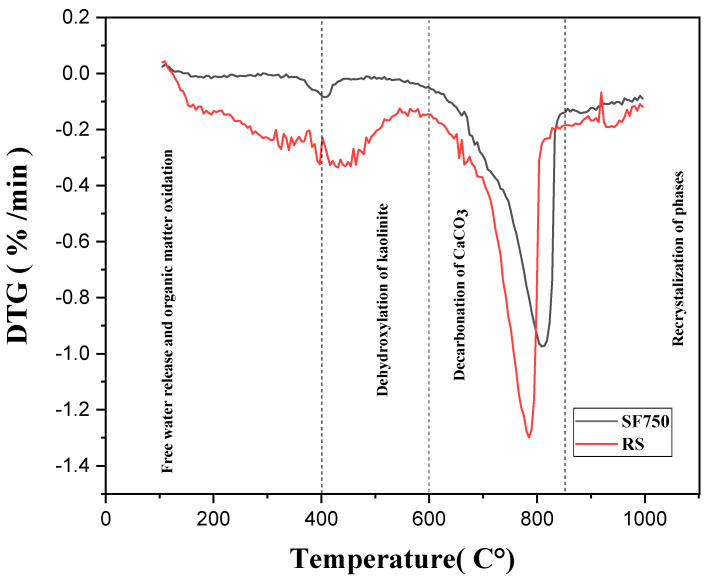
Results of TGA analysis.

The results of the thermogravimetric analysis are presented in Figure 4. The results show three main distinct phases. Up to 600 degrees, the loss of mass corresponds to the elimination of organic matter and the dehydroxylation of clays such as kaolinite. Between 600 and 850 degrees, we observe decalcification and the formation of lime (CaO), and this drop is due to the decomposition of CaCO_3_ into solid CaO and gaseous CO_2_. Finally, beyond that, there is recrystallization and, in some cases, the late dehydroxylation of montmorillonite.

### 2.4. Chemical and Mineralogical Analysis

#### 2.4.1. Chemical Composition FX

The results of the X-ray fluorescence analysis enable us to determine the different oxides present in the three materials used (Table 3). The major oxides are silica (SiO_2_), lime (CaO), alumina (Al_2_O_3_), and iron oxide (Fe_2_O_3_). In addition, minor oxides such as MgO, Na_2_O, SO_3_, and ZnO are detected, and their presence could have an impact on the hydration and properties of the mortar. Chemical composition shows that the content of four main oxides (SiO_2_ + CaO + Al_2_O_3_ + Fe_2_O_3_) in the calcined sediments is equal to 74.1%, which is in accordance with the ASTM C 618 standard [47] specification for coal fly ash and raw or calcined natural pozzolans to be used in concrete. According to this specification, the calcined waste should have the following composition: SiO_2_ + Al_2_O_3_ + Fe_2_O_3_ ≥ 70% and 15% < CaO < 20%.

#### 2.4.2. X-ray Mineralogy

The mineralogical analysis corresponds to the identification of the mineral phases present. The results show the dominant presence of quartz (SiO_2_) and calcite (CaCO_3_) but also other phases such as illite or kaolinite.

**Figure 5:**
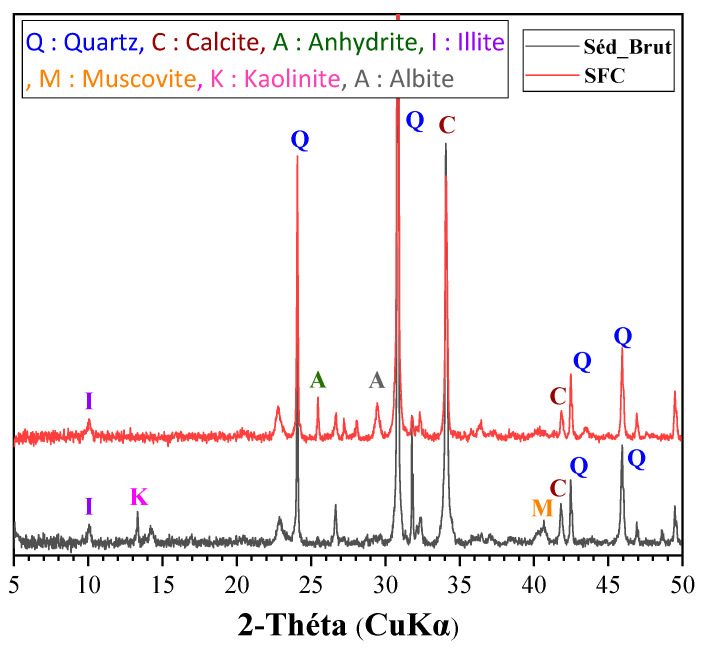
Results of XRD analysis.

The results of the XRD analysis performed on the sediments treated with the raw materials are presented in Figure 5. They also indicate important crystalline modifications with either the disappearance or the appearance of new peaks. The XRD clearly shows a decrease in the appearance of crystalline phases such as calcite due to the decarbonization phase. Furthermore, clay phases like kaolinite are transformed into reactive metakaolin. Moreover, additions with a high calcite (CaCO_3_) content would enhance the hydration of alite (C_3_S) [48] but are strongly unfavorable for C3A [49]. This effect is all the more accentuated as the calcite content is high and the granularity fine.

## 3. Study of Formulation and Characterization of Mortars

### 3.1. Formulation

In order to evaluate the flexural and compressive strength of the mortars, prismatic specimens of size 4 × 4 × 16 cm were prepared according to [34]. The specimens were prepared by partially substituting the calcined sediment with cement with different percentages: 0%, 15%, 20%, and 30%. The rate of mixing water was kept constant for all batches: W/C = 0.5. Prismatic test specimens were taken to study compression and bending tests over 2, 7, 14, 28, and 90 days, respectively. For corrosion potential measurements, cylindrical mortars were prepared using a 5.6 cm diameter by 11.2 cm cylindrical mold in accordance with ASTM C470/C470M-15 [50]. A total of 12 reinforced mortar samples were cast with the same proportions mentioned above. Prior to casting the mortars, ribbed steel bars (high bond) with a diameter of 8 mm and a length of 100 mm were prepared. For each bar, two parts were considered: one measuring 50 mm in length was protected by a resin while the other was exposed to corrosion after being placed in the mold. In addition, 5 × 5 × 5 cm^3^ cubic-shaped mortars were prepared in order to measure the mass variation and the evolution of the compressive strength. Table 4 shows the composition of each mortar. Initially, the samples were placed in a temperature-controlled room with an average temperature of 20 ± 1 °C. They were demolded after 24 h and cured in conditioned water at 20 ± 1 °C until the age specified for the tests. In substitution, the densities of cement and calcined sediments were determined by volume substitution.

### 3.2. Compressive Strength of Mortars

**Figure 6:**
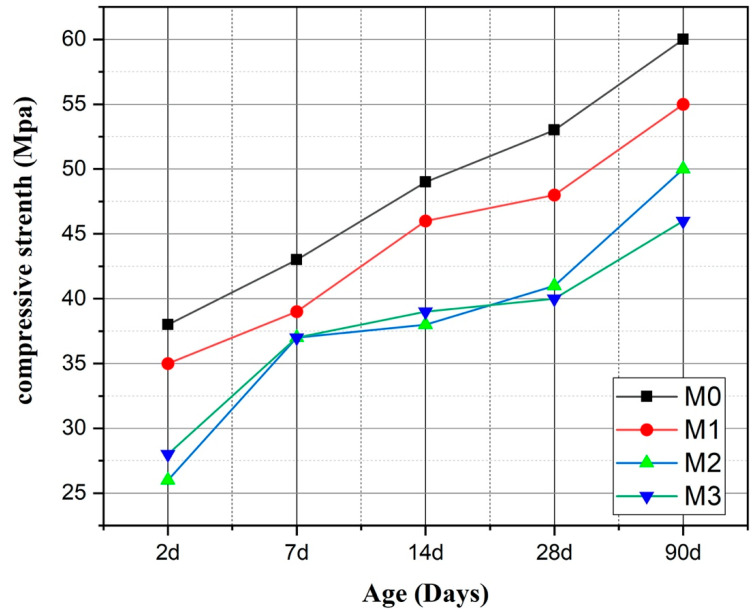
Compressive strength of mortars.

The results of the evolution of the compressive strength of the formulated mortars are presented in Figure 6. The results illustrated here indicate a linear increase in compressive strengths up to 90 days for the different types of mortars. The strengths decrease with the increasing substitution rate. At 90 days, the compressive strengths of M0 and M1 are 60.03 MPa and 55.05, respectively, which means that M1 has a similar overall performance to M0. This suggests that a 15% substitution seems optimal. It should be noted that the presence of calcined sediment creates additional strength, which may be related to certain physical and chemical activities. These results seem to underline the fact that the presence of sediment has an impact on the behavior of the mortar, as determined by [26,27,31].

### 3.3. Flexural Strength of Mortars

**Figure 7:**
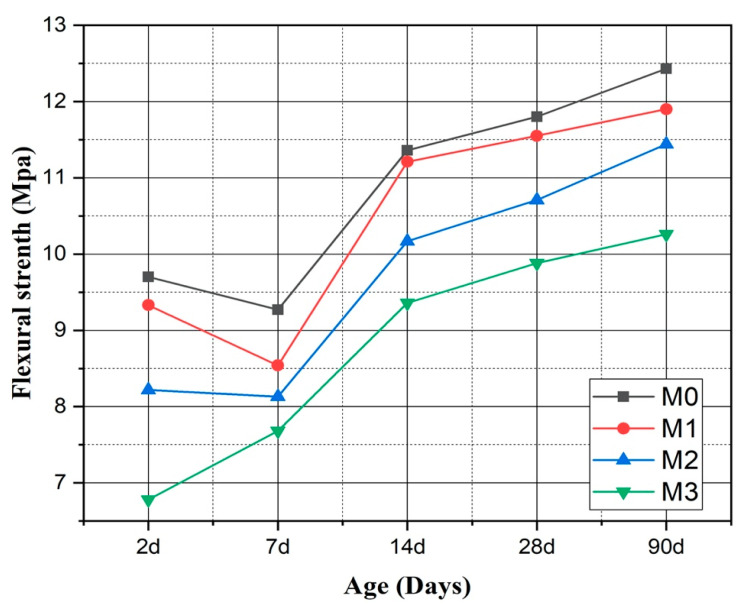
Flexural strength of mortars.

Figure 7 clearly shows the effect of flexural strength on the mixed mortar samples. It is easy to observe a steady increase in flexural strength over time for all mortars and no decrease in flexural strength after 7 days from the start of experiment. Our study shows that the addition of calcined sediment beyond 7 days has a limited impact on flexural strength, regardless of the replacement rate.

### 3.4. Microstructural Study

The characterization of porosity by mercury intrusion represents an important study of the internal structure of mortars. This study, as an overall result (total porosity), provides an index of material quality correlated to compressive strength and also the durability index [51]. The mercury pressure range reaches 30,000 psi (206 MPa) using this method. 

**Figure 8:**
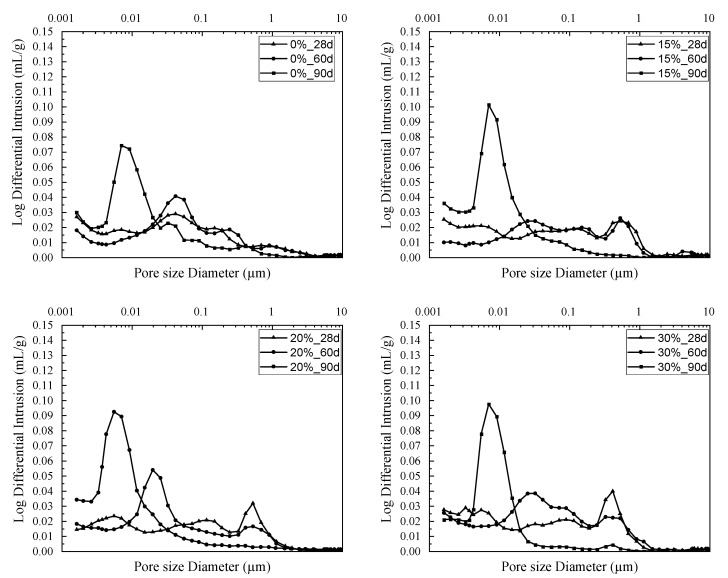
Evolution of pore size distribution.

A porosity study for the four mortars was carried out in order to determine the influence of the added sediments on the properties of the pore network. This parameter determines the main physical–chemical and mechanical properties of a mortar [26]. The samples were subjected to a porosity measurement after a 60-day cure, for 28-, 60-, and 90-day maturities. The results are presented in Figure 8.

Two effects are discussed in this porosity analysis, namely, the evolution of porosity over time and the addition of sediment to the cementitious matrix. For all mix compositions (i.e., incorporating 0, 15, 20, and 30% sediment), a refinement of pore size was observed over time. That is, the more advanced the hydration kinetics, the finer the pore size. This phenomenon is explained by the partial filling of the pores by hydration products. The same observation was made by Kourtaa et al. in their study of lime–pozzolan mixtures (natural and artificial) [52]. The hydration products formed in these types of mixtures are based on lime or cement, with or without mineral addition, filling the pores and at the same time densifying the granular skeleton, which then results in a change in compressive strength. Like fly ash or blast-furnace slag, dredged sediments have also been used as a mineral addition to reduce the use of cement while increasing the mechanical strength and durability of concrete [27,53,54]. They can act as fillers in the cementitious matrix, reducing void volume and increasing overall compactness and density. The ratios studied here have no significant effect on the evolution of pore diameters, since they occupy an equivalent volume which remains negligible for the analysis of mortar porosity.

### 3.5. Resistance to External Sulfate Attack

**Figure 9:**
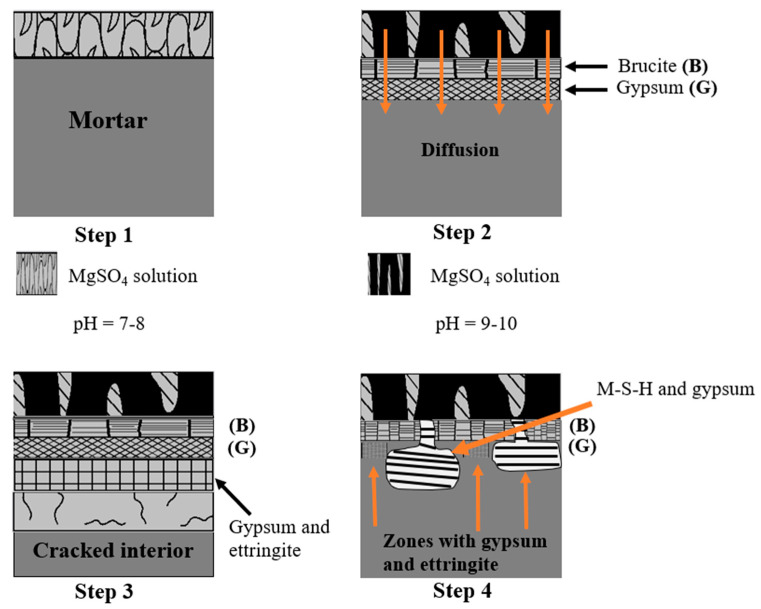
Proposed mechanism of magnesium sulfate attack [56].

The tests used to study the resistance of concrete to sulfate attack are quite varied [55]. The mechanism of external sulfate attack (ESA) on a cementitious material associated with Mg^2+^ follows the following four steps, presented in Figure 9.

First step: The initial stage is initiated by the leaching of portlandite (CH), whereby sulfate (SO_4_^2−^) and calcium ions (Ca^2+^) combine to form secondary gypsum and CSH2 ions. In addition, magnesium ions (Mg^2+^) react with hydroxide ions (OH^−^) to produce brucite (Mg(OH)_2_). The solubility of brucite is very low, around 0.01 g/L, and its presence lowers the pH of the interstitial solution to values close to 10.5, subsequently inducing the decomposition of CSH [57].

(1) CH+MS+2H→CSH2+MH

Second step: The secondary gypsum CSH2 resulting from Equation (1) reacts with the tricalcium aluminates C_3_A to form the secondary ettringite according to Equation (2), a highly expansive product [58].

(2) C3A+3CSH2+26H20→C6AS3H32

Third step: During the consumption of the portlandite (CH), the pH of the interstitial solution drops, which triggers the decalcification of the C-S-H. Then, the ions (Mg^2+^) and (SO_4_^2−^) react to produce gypsum, brucite (B), and silica gel (S2H), as shown in Equation (3) [59,60,61].

(3) CxSyHz+xMS+(3x+0.5y−z)H→xCSH2+xMH+0.5yS2H

Fourth step: The brucite formed (B) reacts with hydrosilicates (S2H), producing hydrated magnesium silicate (M-S-H), which has no binding properties and leads to the disintegration of the paste (Equation (4)) [31].

(4) 4MH+SHn→M4SH8.5+(n−4.5)H

In this study, immersion tests in a magnesium sulfate (MgSO_4_) solution were carried out according to ASTM C1012-04 (2004). The pH of the sulfated solution must be between 6 and 8 and the solution must be renewed every week, which requires considerable quantities of magnesium sulfate. For this purpose, we adopted Mehta’s method [62], which recommends the correction of the solution already used by adding a quantity of sulfuric acid (0.1 N H_2_SO_4_) until the pH of the starting solution is reached (between 6 and 8). The correction is made daily during the first 2 weeks of the test and is then carried out weekly. The solutions are renewed every 20 days.

#### 3.5.1. Visual Inspection

**Figure 10:**
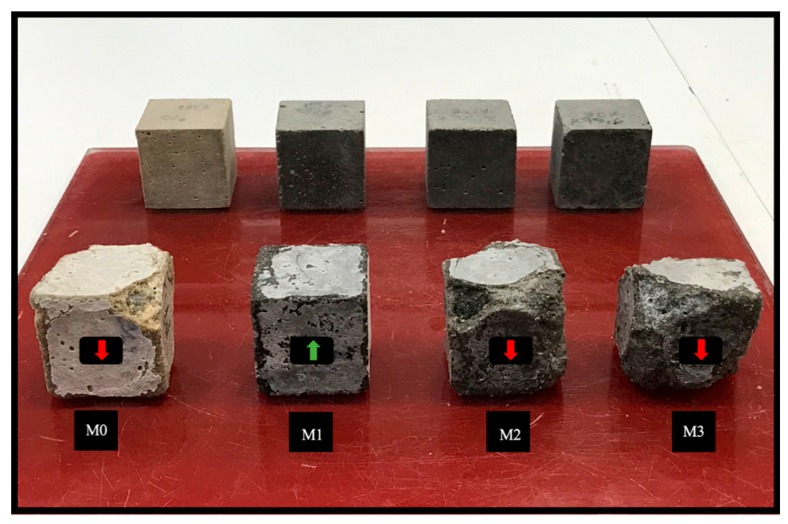
Condition of the mortar samples before and after 360 days immersion 5% MgSO_4_ solution. It can be seen that the M1 mortar has better resistance, which is why it has been marked with a green arrow, unlike the other mortars, which have low resistance and are therefore marked with red arrows.

Visual inspection of the samples was performed every 20 days for 360 days, with the condition of the mortars after 360 days presented in Figure 10. Samples M2 and M3 showed the first signs of corner degradation followed by cracks along the edges, and then, by the end of the follow-up, indicated serious surface damage. Limited deterioration was observed in the corners of sample M0, while for mortar M1, no degradation was observed. In general, the first sign of attack was the deterioration of the corners and edges of the mortars, accompanied by the formation of a whitish layer on the outer surface of the samples which characterizes the onset of gypsum formation.

#### 3.5.2. The Variation of Mass

**Figure 11:**
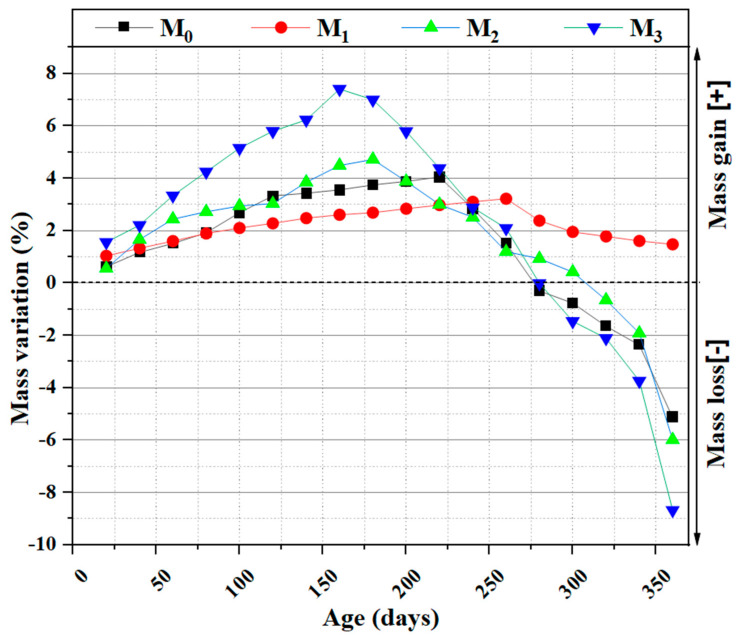
Mass variation of concrete in the 5% MgSO_4_ solution.

The results of mass changes as a function of exposure time in sulfates for different temperatures are presented in Figure 11. All mortars showed an increase in mass followed by a significant loss of mass except for M1, which showed no loss of mass. Samples M0, M2, and M3 indicated a mass loss of 5.14%, 6%, and 8.68%, respectively. The increase in mass observed in the samples exposed to sulfates is mainly due to the reactions between portlandite (CH) and MgSO_4_ to give two products, namely, secondary gypsum (CaSO_4_, 2H_2_O) and brucite Mg(OH)_2_, which are formed on the surface of samples [63]. This increase in mass is also due to the hydration process, which is not yet complete [64]. Other research justifies the increase in mass in the case of the MgSO_4_ attack through the formation of brucite, which is a very poorly soluble product with a low pH [65]. The products that result from this reaction are ettringite and gypsum. The formation of ettringite is related to the increase of eight times the initial volume [66] until the needlelike crystals have no space to grow in the pores. Mass measurements show that the 15% sedimentation of the cement (M1) delays the effect of deterioration due to magnesium sulfate.

#### 3.5.3. The Evolution of Compressive Strength

**Figure 12:**
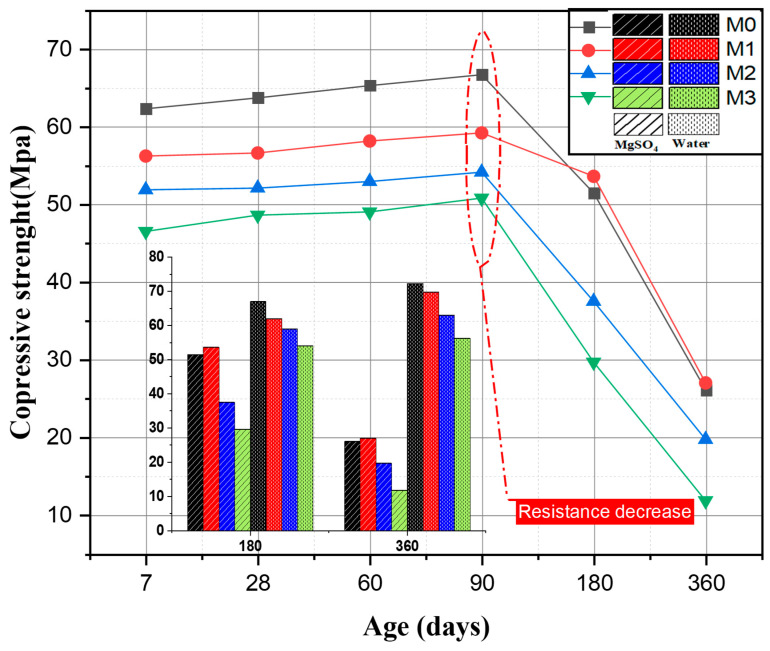
Compressive strength of concrete in the 5% MgSO_4_ solution, and the difference in strength between mortars immersed in MgSO_4_ and water solutions.

Compressive strengths were monitored on 5 × 5 × 5 cm specimens immersed in the magnesium sulfate solution. Figure 12 shows the effect of the partial substitution of OPC cement by calcined sediment SF750 on the evolution of the compressive strength of mortars immersed in a 5% magnesium sulfate solution. Several studies use compressive strength to evaluate external sulfate attacks [67,68,69,70,71,72,73,74]. In their research, Kamile Tosun-Felekoglu [75] studied the effect of sulfate attack on cementitious materials, which was evaluated by measuring the decrease in compressive strength. Pipilikaki [76] also used the loss of compressive strength of mortars to evaluate sulfate damage. The results in Figure 12 indicate an increase in compressive strength for all mortars with up to 90 days of immersion. According to several studies, this increase prior to falling at a certain age is due to the formation of ettringite and gypsum that fill the micropores, leading to a dense structure. Beyond a certain age, the formation of these expansive products causes the destruction of the hardened cement paste and its cracking, which negatively affects the mechanical characteristics of the concrete [77,78]. After 360 days, the M0, M1, M2, and M3 mortars show a significant loss of strength of 60.94%, 54.47%, 63.71%, and 76.82%, respectively. This is in accordance with the studies carried out by Binici [67] and Lee et al. [70], who indicated that magnesium sulfate attack leads to a decrease in compressive strength. 

Histogram bars shows the results for compressive strength at 180 and 360 d. It can be seen that magnesium sulfate soaking weakens the mechanical strength of all mortars compared with mortars that have not undergone soaking. This phenomenon can be explained by the production of expansive products such as secondary ettringite.

The compressive strength results confirm that M1 has a better strength than mortars after 360 days of immersion in MgSO_4_.

### 3.6. Measurement of Corrosion Potential

The measurement of corrosion potential or electrode potential by means of a corrosimeter is one of the most frequently used techniques in the field of nondestructive testing in civil engineering. This method is used to determine the state of corrosion of steel in concrete. Recommendations have been published by ASTM (C876-9) [79] and RILEM TC154-EMC [80].

Reinforced mortar specimens prepared for measuring the evaluation of reinforcement corrosion were first cured in water for 90 days and then partially immersed in a 5% MgSO_4_ solution to allow the bar embedded in the mortar to corrode. Corrosion monitoring of the rebar continued for 360 days, during which the corrosion potentials (i.e., half-cell potential) were regularly measured. The corrosion potentials were measured according to the standard [80] using a saturated Cu/CuSO_4_ copper sulfate electrode. The half-cell potential (HCP) is an effective method that has been used by many researchers around the world [81,82,83]. Table 5 provides guidelines for evaluating corrosion activity on standard copper/copper sulfate half-cells.

**Figure 13:**
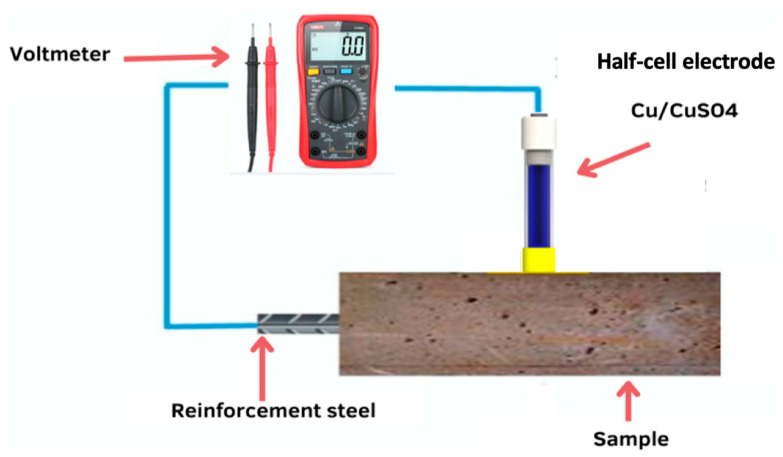
Schematic test setup of the reinforced mortar HCP measurements.

Figure 13 shows a schematic of the HCP measurement setup. According to [79], a digital voltmeter was used to read the potential difference between the external reference electrode and the steel bar. In this study, copper/copper sulfate was used as the reference electrode. If the concrete surface is too dry, it will need to be prewetted. In order to obtain consistent readings, a centerline was drawn on the mortar surface with three measurement points. The potential values for these three points were recorded from the voltmeter for each mortar.

**Figure 14:**
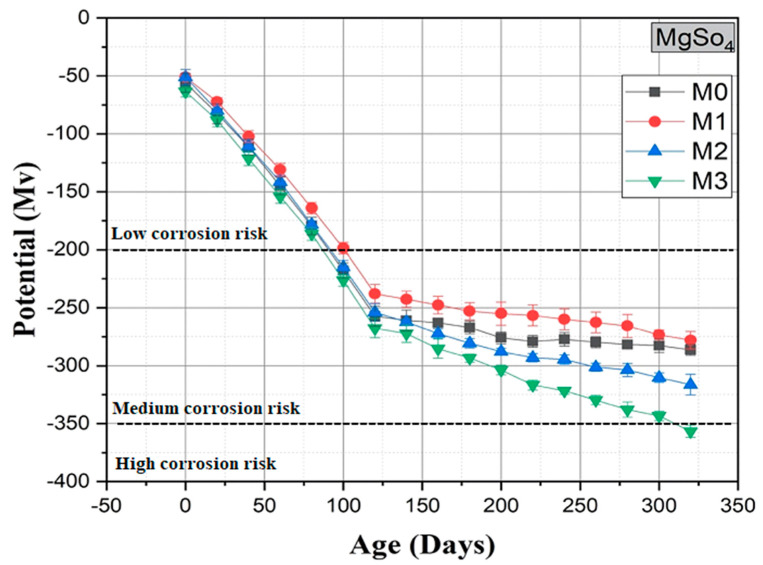
Evolution of potential in the 5% MgSO_4_ solution.

The corrosion potential curves of the four mixtures as a function of time are shown in Figure 14. Corrosion potential curves were used to evaluate the corrosion onset time of steel bars using the standard [79]. A corrosion potential of −350 mV was used as the threshold for corrosion initiation. According to this standard, if the measured corrosion potential is below −270 mV, there is a 90% probability that corrosion will start. No corrosion initiation of the steel for the M0, M1, and M2 mortars was observed, while for the M3 mixture, a probability of corrosion was observed from 310 days. When 15% SF 750 was added to the OPC mixture (M1), the corrosion resistance was significantly improved and even higher than that of the reference mortar (M0), as shown in the corrosion potential diagram (Figure 14).

It can be observed that in the presence of SF 750 with a substitution total of up to 20%, even after 300 days of exposure to a 5% MgSO_4_ solution, the corrosion of the steel does not commence (i.e., the corrosion potential is less negative than the threshold limit of −350 mV). These results confirmed the beneficial effect of the incorporation of SF 750, making the mortar more resistant to corrosion of the reinforcement embedded in the mortar.

Finally, experimental results showed that mortars incorporating up to 15% SF 750 showed satisfactory durability for 365 days of exposure to a 5% MgSO_4_ solution, while in the literature, it was noted that the substitution of 10% to 12.5% of calcined sediment is beneficial for the mechanical properties and durability of concrete [26,27].

## 4. Conclusions

This study was conducted to investigate the effects of the partial replacement of ordinary Portland cement (OPC) with calcined flash sediments (SF 750) on the mechanical and durability properties of mortar. This study showed that the use of SF 750 as a mineral addition is beneficial and promotive. The conclusions drawn from the results of this study are as follows:

The mortar mix with 30% OPC replaced by SF 750 indicated the lowest compressive strength. The M1 has a compressive strength almost similar to that of the M0, with a slight superiority in the latter.

The resistance of the M1 mortar mix to sulfate attack was higher than that of other mixes.

A 30% replacement of cement by calcined flash sediment has a negative effect on corrosion in the sulfate environment.

Replacing 15% of the cement with the calcined flash sediment resulted in a reduction in the corrosion rate (decrease in corrosion rate) in an environment containing 5% MgSO_4_ for 365 days.

Finally, it can be concluded that the use of 15% SF 750 as a mineral addition has a very beneficial effect on the overall performance of concrete, including strength and durability. This study, therefore, resulted in the formulation of a mortar mix with an optimal binder combination, 75% OPC–15% SF 750, that can be produced with technical, economic, and environmental benefits.

## Figures and Tables

**Table 1:** Nomenclature of materials used in this study.

Materials	Nomenclature
Ordinary Portland cement CEM I 52.5 N	OPC
Raw sediment	RS
Sediment calcined flash at 750 °C	SF 750
Witness mortar	M0
Mortar with 15% sediment	M1
Mortar with 20% sediment	M2
Mortar with 30% sediment	M3

**Table 2:** Physical properties of materials.

Materials	CEM I 52.5 N (OPC)	Raw Sediment (RS)	Flash Calcined Sediment (SF 750)	Natural Sand (NS)
Density (g/cm^3^)	3.15	2.48	2.65	—
BET (m^2^/g)	1.06	7.32	27.49	—
LOI (%)	1.90	16.10	4.35	—
D10 (μm)	1.02	0.69	1.22	—
D50 (μm)	8.62	3.99	8.03	—
D90 (μm)	27.00	15.84	25.39	—

**Table 3:** Results of FX analysis.

	Na_2_O	MgO	Al_2_O_3_	SiO_2_	P_2_O_5_	SO_3_	K_2_O	CaO	TiO_2_	Fe_2_O_3_	ZnO
RS (%)	0.45	0.75	8.65	33.25	1.36	0.26	1.49	13.10	0.42	3.89	0.17
SF750 (%)	0.63	0.91	10.30	43.45	1.56	0.32	1.84	15.40	0.54	4.95	0.25
OPC (%)	0.79	0.90	5.30	19.54	—	3.61	0.83	64.02	—	3.15	—

**Table 4:** Composition of the different mortar formulations (values given in grams).

Materials	M0	M1	M2	M3
Sand	1350	1350	1350	1350
OPC	450	391.44	371.31	330.09
SF750	—	58.55	78.68	119.91
Water	225	225	225	225

**Table 5:** Predicting corrosion using Cu/CuSO_4_ half-cell [79].

Corrosion Risk	Half-Cell Potential (Versus Cu/CuSO_4_)
Severe corrosion	Less than −500 mV
High corrosion risk (90% probability)	Between −500 mV & −350 mV
Medium corrosion risk (50% probability)	Between −500 mV & −350 mV
Low corrosion risk (10% probability)	Higher than −200 mV

## Data Availability

The data used in this study are available upon request. Researchers interested in accessing the data may contact mouhamadou.amar@imt-nord-europe.fr.
